# Characterization of Remitting and Relapsing Hyperglycemia in Post-Renal-Transplant Recipients

**DOI:** 10.1371/journal.pone.0142363

**Published:** 2015-11-09

**Authors:** Alireza Boloori, Soroush Saghafian, Harini A. Chakkera, Curtiss B. Cook

**Affiliations:** 1 Department of Industrial Engineering, School of Computing, Informatics and Decision Systems Engineering, Arizona State University, Tempe, Arizona, United States of America; 2 Harvard Kennedy School, Harvard University, Cambridge, Massachusetts, United States of America; 3 Division of Nephrology and Transplantation, Mayo Clinic School of Medicine, Scottsdale, Arizona, United States of America; 4 Division of Endocrinology, Mayo Clinic School of Medicine, Scottsdale, Arizona, United States of America; University of Toledo, UNITED STATES

## Abstract

**Background:**

Hyperglycemia following solid organ transplant is common among patients without pre-existing diabetes mellitus (DM). Post-transplant hyperglycemia can occur once or multiple times, which if continued, causes new-onset diabetes after transplantation (NODAT).

**Objective:**

To study if the first and recurrent incidence of hyperglycemia are affected differently by immunosuppressive regimens, demographic and medical-related risk factors, and inpatient hyperglycemic conditions (i.e., an emphasis on the time course of post-transplant complications).

**Methods:**

We conducted a retrospective analysis of 407 patients who underwent kidney transplantation at Mayo Clinic Arizona. Among these, there were 292 patients with no signs of DM prior to transplant. For this category of patients, we evaluated the impact of (1) immunosuppressive drugs (e.g., tacrolimus, sirolimus, and steroid), (2) demographic and medical-related risk factors, and (3) inpatient hyperglycemic conditions on the first and recurrent incidence of hyperglycemia in one year post-transplant. We employed two versions of Cox regression analyses: (1) a time-dependent model to analyze the recurrent cases of hyperglycemia and (2) a time-independent model to analyze the first incidence of hyperglycemia.

**Results:**

Age (*P* = 0.018), HDL cholesterol (*P* = 0.010), and the average trough level of tacrolimus (*P*<0.0001) are significant risk factors associated with the first incidence of hyperglycemia, while age (*P*<0.0001), non-White race (*P* = 0.002), BMI (*P* = 0.002), HDL cholesterol (*P* = 0.003), uric acid (*P* = 0.012), and using steroid (*P* = 0.007) are the significant risk factors for the recurrent cases of hyperglycemia.

**Discussion:**

This study draws attention to the importance of analyzing the risk factors associated with a disease (specially a chronic one) with respect to both its first and recurrent incidence, as well as carefully differentiating these two perspectives: a fact that is currently overlooked in the literature.

## Introduction

Hyperglycemia is a well-described complication following solid organ transplantation [1–3]. Among patients without a prior history of diabetes mellitus (DM), hyperglycemia that either persists after transplant, or which resolves but later recurs and persists, is termed new onset diabetes after transplant (NODAT). Hyperglycemia and NODAT are strong predictors of graft failure and cardiovascular mortality occurring commonly after solid organ transplant [1–3]. The occurrence of hyperglycemia or development of NODAT have been attributed to many factors, including (1) immunosuppressive drugs and their diabetogenic effects, (2) other demographic and medical-related risk factors, and (3) inpatient hyperglycemic conditions.

Regarding the first factor, Table 1 summarizes studies on the diabetogenic effect of anti-rejection agents (e.g., tacrolimus, sirolimus, cyclosporine, glucocorticoids, and steroid) with respect to different solid organ transplantations (e.g., kidney, liver, and pancreas). The main insights from this literature are related to: (1) the efficacy of a drug in preventing organ rejection while imposing less risk for hyperglycemia or NODAT, (2) the relative benefits/side effects of two or more drugs when compared with each other, and (3) the potentials of drugs when switching from one therapy to another.

**Table 1:** Classification of literature based on diabetogenic effect of immunosuppressive drugs.

**Drug Type**	**Organ Type**	**Selected References**
Tacrolimus	Kidney/Liver	[4–18]
Sirolimus	Kidney/Liver	[17, 19–26]
Cyclosporine	Kidney	[18, 27–37]
Glucocorticoids	Kidney/Pancreas	[18, 38–44]
Steroid	Kidney/Pancreas	[45–48]

In addition to immunosuppressive drugs, the literature has analyzed other demographic or medical-related risk factors to establish possible statistically significant associations with hyperglycemia and NODAT (Table 2). The majority of the literature in this stream attempts to (1) derive associations between risk factor(s) and a continuous variable (linear regression models) that represents hyperglycemia/NODAT status (e.g., blood glucose level measured by hemoglobin A1c and fasting plasma glucose tests), (2) demonstrate the same effect for a categorical variable (i.e., whether a patient suffers from hyperglycemia or not, at a specific point of time) by applying logistic regression models, or (3) discuss the probability of survival from hyperglycemia/NODAT at a single point of time (Cox regression models).

**Table 2:** Classification of literature based on the impact of risk factors on hyperglycemia and NODAT.

**Risk Factor**	**Organ Type**	**Selected References**
Age	Kidney/Liver	[49–57]
Gender	Kidney/Liver	[53, 54, 57–62]
Race/Ethnicity	Kidney	[49, 52, 54, 57, 63, 64]
BMI	Kidney/Liver	[49–51, 53–57, 64]
Cadaveric organ	Kidney/Liver	[50, 51, 53–55, 57]
Hepatitis C Virus	Kidney/Liver	[49, 51, 53, 55, 57, 64]
Hypertension	Kidney	[52, 55, 64–66]
Diabetes History	Kidney	[49, 53, 57, 64, 66]

Furthermore, recent evidence indicates that hyperglycemia occurring in the immediate post-transplant period (i.e., during the post-operative hospital stay) is also associated with NODAT [67, 68].

In spite of all these efforts, none of these factors (immunosuppressive drugs and their diabetogenic effects, demographic and medical-related risk factors, and inpatient hyperglycemic conditions) have been analyzed with respect to the time course of post-transplant complications. Specifically, one critical aspect that is overlooked by the literature is an understanding and analysis of *remitting and relapsing* hyperglycemia in post-solid organ transplant recipients. Such an understanding can be critical because (1) the insights gained can be quite different from those previously known for the incidence of hyperglycemia and (2) these insights can be extended to other chronic diseases with the possibility of remitting and relapsing, such as cancer and multiple sclerosis. To the best of our knowledge, this is the first study analyzing the first and recurrent incidence of hyperglycemia. In particular, utilizing a population of renal transplant recipients who had no history of DM before transplantation, we undertake a set of analyses to determine which contributing factors are significantly associated with the first incidence, and which ones are significantly associated with the recurrent incidence.

## Materials and Methods

### Study Cohort

After obtaining Mayo Clinic Institutional Review Board (Mayo Clinic IRB) approval (Continuing Review #: PR13-004295-01) and written informed consent from all participating patients, this study conducts an analysis of 292 patients who underwent a renal transplant between 1999 and 2006 in Mayo Clinic Arizona, and who had no history of DM prior to surgery. Briefly, all patients were monitored at the time of transplant as well as month 1, 4, and 12 post-transplant. The available data included (1) demographic data such as age, race, and gender, (2) baseline patient characteristics including body mass index (BMI), blood pressure (BP), total cholesterol (Chol), high-density lipoprotein cholesterol (HDL), low-density lipoprotein cholesterol (LDL), uric acid (UA), and triglyceride (TG), (3) type of immunosuppressive drugs and diabetes medications that were used by the patients, (4) trough level of tacrolimus (as the main immunosuppressive drug used in this study), and (5) results of fasting plasma glucose (FPG) and Hemoglobin A1c (HbA1c) tests as measures of glycemic control. All major abbreviations used in this study are explained in Table 3.

**Table 3:** Description of abbreviations used in this study.

**Abbreviation**	**Description**
HG	Hyperglycemia
FPG	Fasting plasma glucose
HbA1c	Hemoglobin A1c
*C*_0_	Trough level of tacrolimus
BMI	Body mass index
BP	Blood pressure
Chol	Total cholesterol
HDL	High-density lipoprotein cholesterol
LDL	Low-density lipoprotein cholesterol
TG	Triglyceride
UA	Uric acid

### Definitions

NODAT was defined as HbA1c ≥ 6.5%, or FPG ≥ 126 mg/dL, or the requirement of diabetes medications (e.g., insulin or oral agent) after patient discharge from hospital [67, 68]. We apply this criteria to determine the incidence of post-transplant hyperglycemia, which may happen just once or for multiple times (recurrent). We refer to either of these conditions as instances of *remitting and relapsing* hyperglycemia.

### Statistical Methods

We now explain the statistical inference methods we employed to analyze the effects of immunosuppressive drugs, the corresponding risk factors, and the inpatient period conditions on the first and recurrent incidence of post-transplant hyperglycemia. The statistical models used were: (i) The Cox regression model with time-dependent covariates, which measures the *proportional hazard* imposed on the response variable (hyperglycemia incidence) by covariates that change over time. For example, the BMI of a patient may change as his/her weight changes (Chol, HDL, and LDL are some other examples of such covariates). As another example, whether the patient uses an immunosuppressive drug at a specific time or not can be considered as a time-dependent covariate. Therefore, we sought to fully comprehend the effect of these changing behaviors on the recurrent incidence of hyperglycemia. (ii) Cox regression model with time-independent covariates, which measures the proportional hazard imposed on the response variable (the first incidence of hyperglycemia) by covariates at the time of the first incidence of hyperglycemia. (iii) Kaplan-Meier survival analysis to characterize the cumulative probability of experiencing hyperglycemia over time.

The statistical analyses also include *multiple imputations by chained equations* (MICE) [69], which we used to replace some missing data (with the prevalence of less than 10% in our data set) with validated values. We conducted all statistical analyses by using the R computing package.

## Results

### Demographic and Baseline Characteristics of Patients

Among 407 patients in the study cohort, there were 115 patients with the history of diabetes. The remaining 292 patients had no indication of diabetes prior to or at the time of their transplants. The average age of patients who had no diabetes before transplant was 49.7 years, while those who had diabetes before had the average age of 56. Table 4 summarizes the demographic data along with some other baseline characteristics of patients.

**Table 4:** Demographic and baseline characteristics of patients at the time of transplant.

**Characteristics**	**Diabetes History (n = 115)**	**No Diabetes History (n = 292)**
Age (year)	56.0 ± 10.4^a^	49.7 ± 14.6
Gender: Male (%)	61.74	56.16
Race: White^b^ (%)	59.13	75.34
BMI (kg/m^2^)	28.7 ± 5.4	27.0 ± 5.6
Donor: Live^c^ (%)	52.17	67.47
Pre-transplant FPG (mg/dL)	143.8 ± 52.3	92.8 ± 11.3
Pre-transplant HbA1c (%)	6.9 ± 1.5	5.5 ± 0.3
Pre-transplant UA (mg/dL)	6.3 ± 2.3	6.6 ± 2.1
Pre-transplant Chol(mg/dL)	183.0 ± 47.4	181.9 ± 46.0
Pre-transplant HDL (mg/dL)	50.6 ± 16.0	50.6 ± 16.0
Pre-transplant LDL (mg/dL)	94.2 ± 33.0	93.9 ± 34.8
Pre-transplant TG (mg/dL)	191.2 ± 94.7	179.0 ± 87.7

^a^ mean ± standard deviation,

^b^ versus non-white (including Native American, Hispanic, and Black races),

^c^ versus cadaveric.

### Incidence of Hyperglycemia

Regarding the definition of remitting and relapsing hyperglycemia, Table 5 summarizes different hyperglycemic states that can occur after renal transplantation. Therefore, 79 (27.06%) patients experienced *remitting and relapsing* post-transplant hyperglycemia (and hence the hyperglycemia for the first time). Among these patients, 19+3+1+24 = 47 patients experienced hyperglycemia multiple times, while 20+11+1 = 32 had it just once. As an example of the potential remitting and relapsing nature of post-transplant hyperglycemia, there are 11 patients who developed hyperglycemia at 4 months, which resolved at 12 months.

**Table 5:** Percentage of patients satisfying the criteria.

**Time**	**Having the criteria?**							
Month 1	No	Yes	No	No	Yes	Yes	No	Yes
Month 4	No	No	Yes	No	Yes	No	Yes	Yes
Month 12	No	No	No	Yes	No	Yes	Yes	Yes
# of patients (%)	213 (72.95)	20 (6.85)	11 (3.77)	1 (0.34)	19 (6.51)	3 (1.03)	1 (0.34)	24 (8.22)

### Summary of Immunosuppressive Treatment Regimens

This section sheds light on information about main immunosuppressive medications that have been considered for this study (tacrolimus, steroid, and sirolimus). As mentioned before, we focus on 292 patients with no prior history of diabetes.

#### Tacrolimus

**Fig 1:**
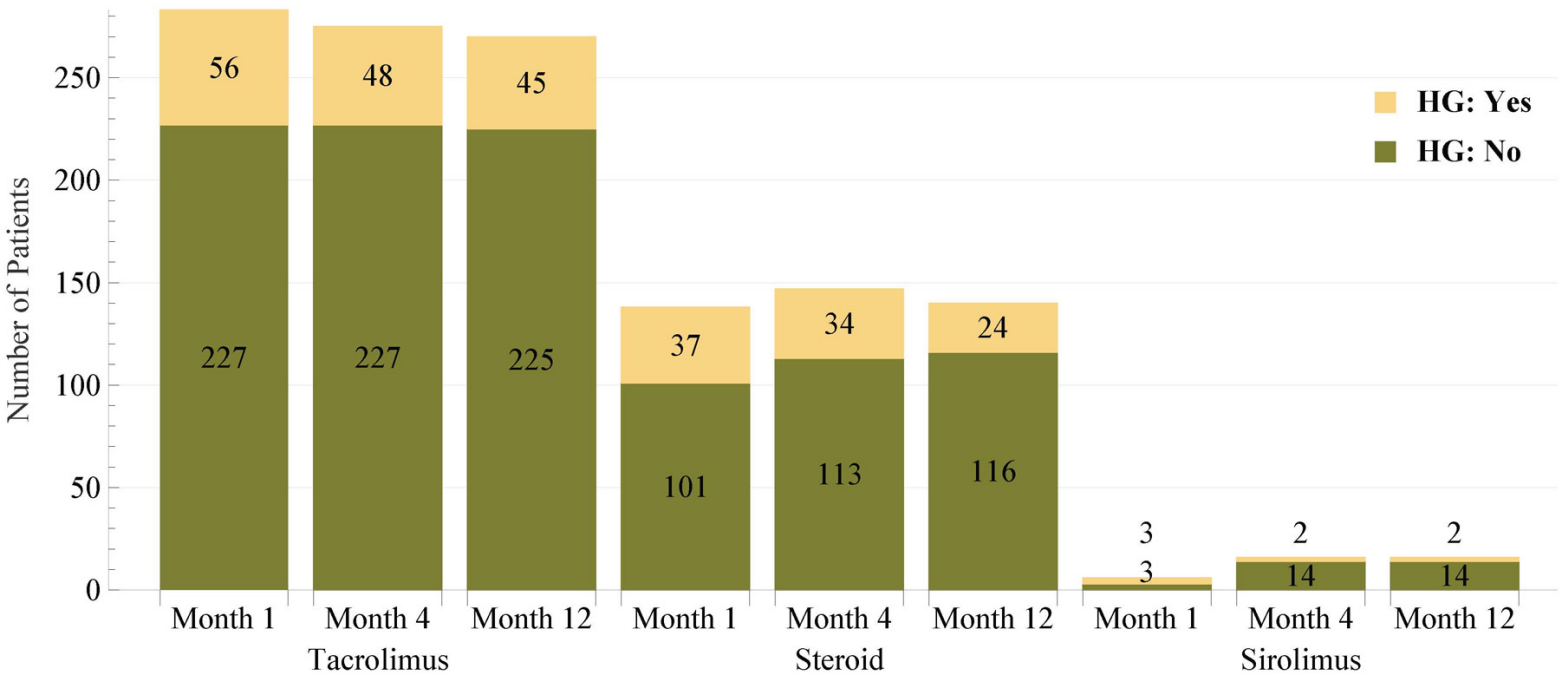
Number of patients who used immunosuppressive drugs at months 1, 4, and 12. Such patients are further classified as having hyperglycemia (HG) or not at that specific time points.

Tacrolimus (Prograf) is the main immunosuppressive drug utilized in this study. Fig 1 (the first three columns) demonstrates the number of patients at month 1, 4, and 12 using tacrolimus, which include 283, 275, and 270 patients (out of 292 patients), respectively. As our primary interest in this study is to analyze the incidence of hyperglycemia, we further classified patients in terms of whether they experienced hyperglycemia at a specific time or not, and Fig 1 reveals this information as well.

Another important point regarding tacrolimus is the dosage goals and achieved levels at different points of time. Tacrolimus goals are adjusted to avoid toxicity and to the lowest dose possible to avoid rejection per clinical standards of care. This is a standard clinical practice and is based on individual response and pharmacokinetics. Table 6 summarizes this information. It should be noted that the achieved levels of tacrolimus are represented in terms of the average trough level of tacrolimus.

**Table 6:** Tacrolimus goals and achieved levels (average trough level) at months 1, 4, and 12.

**Time point**	**Tacrolimus goal**	**Tacrolimus achieved average trough level**
1 month	10–12 mg/dL	11.88 mg/dL
4 months	8–10 mg/dL	9.59 mg/dL
12 months	6–8 mg/dL	7.83 mg/dL

#### Steroid

Steroid is the second main immunosuppressive drug incorporated in this study. Fig 1 (the second three columns) illustrates the number of patients at month 1, 4, and 12 using steroid, which include 138, 147, and 140 patients (out of 292 patients), respectively. This shows that in comparison with tacrolimus which was used by the majority of patients, fewer patients used steroid. (According to what explained for tacrolimus, the percentages of patients using tacrolimus at months 1, 4, and 12 were 283/292 = 97%, 275/292 = 94%, and 270/292 = 92%, respectively.) Fig 1 also shows the number of patients who used steroid and experienced hyperglycemia.

Steroid is usually prescribed by the following mechanism. If after using induction steroids (which last for up to 5 days post-transplant) a patient has an organ rejection, she will receive a taper dose of steroid (i.e., slow withdrawal). Then, by 1 month post-transplant, the patient will be put on the maintenance regimen of 5 mg daily (which is a low dosage), unless the patient has another rejection(s) later and needs possibly extra dosage of steroid therapy. To this end, we observed the following from the data set: (1) Among the 292 patients, only 20 patients had organ rejection at month 1, and hence, had to use a taper dose of steroid at this month. Therefore, there remained 272 patients who had no rejection during month 1. (2) Among 20 patients at month 1, 4 patients at month 4 and 1 patient at month 12 experienced organ rejection (these were mutually exclusive patients). (3) Among 272 patients at month 1, 5 patients at month 4 and 6 patients at month 12 experienced organ rejection (these were mutually exclusive patients). Therefore, according to the mechanism explained before, it can be concluded that 4+5+1+6 = 16 patients (out of 292) had increased dose of steroid (i.e., more than 5 mg daily) after 1 month post-transplant. Furthermore, as explained before, according to Fig 1, 138, 147, and 140 patients used steroid at months 1, 4, and 12, respectively. Therefore, 138-(4+1) = 133, 147-(4+5) = 138, and 140-(1+6) = 133 patients remained on the regimen of 5 mg daily at months 1, 4, and 12, respectively.

#### Sirolimus

Sirolimus (Rapamune) is the third main immunosuppressive drug incorporated in this study. Fig 1 shows that sirolimus was utilized by a very small proportion of patients.

### Time-Dependent Cox Regression Model: Recurrent Incidence of Hyperglycemia

To address events that may occur repeatedly, such as the repeated occurrence of hyperglycemia, we need to incorporate covariates that change over time (e.g., BMI, BP, etc.). To this end, we employed a Cox regression model with time-dependent covariates and recurrent events, where each event is assumed to occur once a patient meets the criteria defined in Table 5. The performance measure in this model is the *hazard ratio* (HR), such that if mean HR ≥ 1, the corresponding covariate will have a positive effect on the response variable (and vice versa).

According to Table 7 (Part I), induction immunosuppressive agents (thymoglobulin and simulect) and steroids were significantly associated with lower and higher chance of recurrent hyperglycemia, respectively. However, neither using tacrolimus nor its average trough level was significantly associated with the repeated occurrence of hyperglycemia. Therefore, one cannot establish the diabetogenic effect of tacrolimus when hyperglycemia occurs repeatedly. As we will see in the next section, this finding is in a sharp contrast with the case where only the first incident of hyperglycemia is considered.

**Table 7:** Effect of immunosuppressive drugs on hyperglycemia: The results of two statistical inference methods (numbers in bold represent statistically significant covariates at 95% confidence level).

**Covariates**	**Part I: Time-dependent**				**Part II: Time-independent**			
	**Mean HR**	**Lower CI** ^a^	**Upper CI**	**P-value** ^b^	**Mean HR**	**Lower CI**	**Upper CI**	**P-value**
Simulect^c^ (unadj^e^)	0.51	0.274	0.953	**0.035**	0.655	0.299	1.437	0.291
Simulect (adj^f^)	0.267	0.131	0.543	**0.000**	0.444	0.190	1.036	0.060
Thymoglobulin^c^ (unadj)	0.68	0.480	0.950	**0.025**	0.645	0.401	1.038	0.071
Thymoglobulin (adj)	0.658	0.458	0.947	**0.024**	0.640	0.388	1.055	0.080
Avg. *C*_0_ (unadj)	0.993	0.859	1.147	0.924	1.949	1.793	2.120	**0.000**
Avg. *C*_0_ (adj)	0.992	0.859	1.146	0.912	1.982	1.788	2.197	**0.000**
Tacrolimus^d^ (unadj)	0.922	0.434	1.963	0.834	1.285	0.470	3.512	0.625
Tacrolimus (adj)	0.689	0.297	1.601	0.387	1.156	0.397	3.370	0.790
Sirolimus^d^ (unadj)	1.329	0.655	2.694	0.431	0.834	0.305	2.279	0.723
Sirolimus (adj)	1.786	0.852	3.745	0.124	0.810	0.280	2.344	0.697
Steroid^d^ (unadj)	1.230	0.894	1.691	0.204	1.248	0.803	1.939	0.325
Steroid (adj)	1.562	1.131	2.158	**0.007**	1.441	0.900	2.305	0.128

^a^ 95% confidence interval,

^b^ P-values are obtained based on standard Normal distribution,

^c^ An immunosuppressive agent: Induction therapy,

^d^ An immunosuppressive agent: Maintenance therapy,

^e^ Unadjusted (univariate) analysis,

^f^ All adjusted analyses were done based on age, race, gender, BMI, BP, Chol, HDL, LDL, UA, and TG.

### Time-Independent Cox Regression Model: First Incidence of Hyperglycemia

The reason that the diabetogenic effect of tacrolimus cannot be established when hyperglycemia is occurring repeatedly may be due to the fact that tacrolimus dosage is usually reduced with the passage of time after transplant (see Table 6). To test this hypothesis, we analyzed the immunosuppressive effect when hyperglycemia happens for the first time. We used a time-independent Cox regression model, in which only covariates at the time of first occurrence are considered. According to Table 7 (Part II), the average trough level of tacrolimus is significantly associated with a higher chance of first hyperglycemia incident, which implies that the diabetogenic effect of tacrolimus can be established in this case. This observation highlights the importance of differentiating between the first and recurrent incidents of hyperglycemia.

As other observations made in this regard, induction immunosuppressive agents (thymoglobulin and simulect) are significantly associated with lower chance of first hyperglycemia. However, we cannot establish any significant association between using steroid and the first incidence of hyperglycemia. This can be due to the fact that high dosages of steroid were only considered for a small proportion of patients in month 1 post-transplant (see section “Summary of Immunosuppressive Treatment Regimens” for more information).

### Kaplan-Meier Analysis: Hyperglycemia Incidence

The results of time-independent analysis established by the Cox regression model shows the significant association between the average trough level of tacrolimus and the first incidence of hyperglycemia. Here, we aim to use *Kaplan-Meier survival analysis* to calculate the probability of having hyperglycemia obtained from Kaplan-Meier survival curves.

To this end, we consider the main *stratum* based on average trough level of tacrolimus classified as “≤10” and “>10” mg/dL. In order to fully comprehend the effect of these levels on the incidence of hyperglycemia, we conduct unadjusted (univariate) analysis as well as ten adjusted analyses for those risk factors mentioned before. However, to incorporate these risk factors into the Kaplan-Meier survival analysis, they should be discretized in classes, which are shown in Table 8. It should be noted that the classification thresholds for each of these risk factors have been set so as to distinguish the groups in terms of health-related risks (e.g., BMI of 30 kg/m^2^ for obesity). Furthermore, except age, gender, race, and blood pressure, other thresholds have been obtained from [70]. Regarding the blood pressure, if the systolic and diastolic blood pressure are “<120” and “<80” mm Hg, respectively, the patient is normal. Otherwise, the patient has hypertension. These thresholds have been obtained from [71].

**Table 8:** Description of groups formed by risk factors.

**Risk Factors**	**Unit**	**Group 0**	**Group 1**
Age	Years	<50	≥ 50
Gender	—	Female	Male
Race	—	White	non-White
BMI	kg/m^2^	<30 (non-obese)	≥30 (obese)
BP	—	Normal	Hypertension
Chol	mg/dL	<200	≥200
HDL	mg/dL	≥40	<40
LDL	mg/dL	<130	≥130
TG	mg/dL	<150	≥150
UA	mg/dL	<7.3	≥7.3

**Fig 2:**
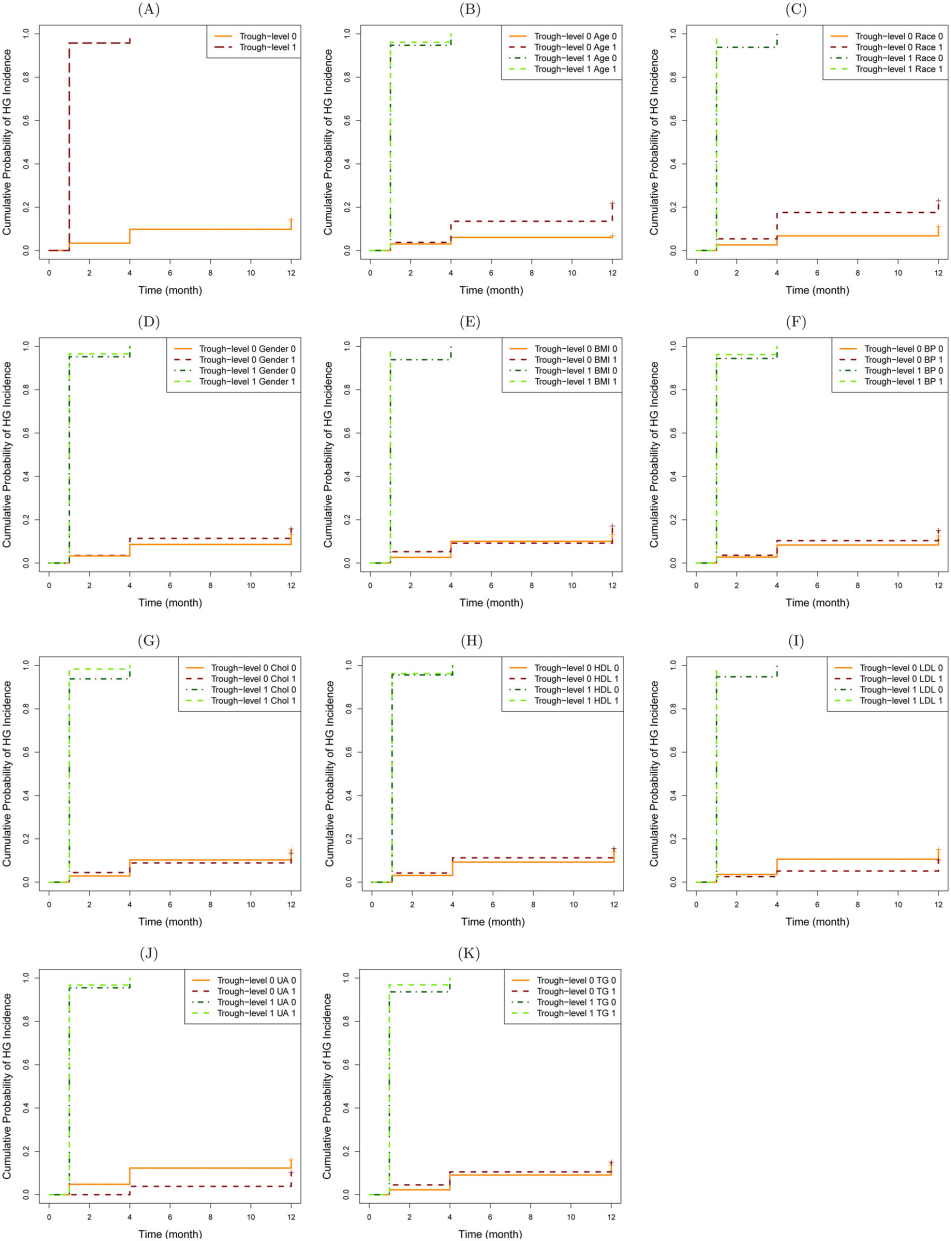
Kaplan-Meier survival curves: Cumulative probability of experiencing hyperglycemia (%) as a result of having different average trough levels of tacrolimus: ≤10 mg/dL vs. >10 mg/dL. In all parts (A)-(K), the *P*-value by the Logrank test is <0.0001. (+ represents censored events.). (A) Unadjusted (univariate) analysis. (B) Adjusted analysis with age. (C) Adjusted analysis with race. (D) Adjusted analysis with gender. (E) Adjusted analysis with BMI. (F) Adjusted analysis with BP. (G) Adjusted analysis with Chol. (H) Adjusted analysis with HDL. (I) Adjusted analysis with LDL. (J) Adjusted analysis with UA. (K) Adjusted analysis with TG.

Fig 2 presents the above-mentioned survival curves. For simplicity, patients with an average tacrolimus trough level of less than or equal to (more than) 10 mg/dL are said to have *Trough-level 0 (1)*. Fig 2A shows that Trough-level 1 patients have significantly higher chance of experiencing hyperglycemia (HG) than Trough-level 0 patients (i.e., Logrank *P* <0.0001). Specifically, almost all of the former group experience HG by month 4 (i.e., probability of experiencing HG ≈ 100%), while the latter group still have about 80% chance of not experiencing HG by year 1. Although we made these observations for the unadjusted (univariate) analysis, the same behavior can be seen for adjusted analyses: the chance of experiencing HG is significantly different (i.e., Logrank *P* <0.0001) across groups formed by different risk factors (see Fig 2B–2K). Furthermore, Trough-level 1 patients with any of the following conditions almost certainly experience HG by month 1: non-White ethnicity, obese (BMI >30 kg/m^2^), and LDL ≥130 mg/dL. Moreover, Trough-level 1 patients with any of the following conditions experience HG by month 1 with a chance not less than 90%: age>50, male, hypertension, Chol ≥200 mg/dL, HDL <40 mg/dL, UA ≥7.3 mg/dL, or TG ≥150 mg/dL.

### Other Risk Factors for the Incidence of Hyperglycemia

We also analyzed the associations of other well-known risk factors for both the first and recurrent incidence of hyperglycemia. To this end, we again applied two types of Cox regression model. The results of these analyses are provided in Table 9. We found that age and HDL were significantly associated with the first incident of hyperglycemia, whereas age, race (non-White), BMI, HDL, and UA were significant risk factors for the recurrent incidence of hyperglycemia.

**Table 9:** Risk factors that affect the incidence of hyperglycemia.

**Risk Factors**	**Part I: Time-dependent**				**Part II: Time-independent**			
	**Mean HR**	**Lower CI^a^**	**Upper CI**	**P-value^b^**	**Mean HR**	**Lower CI**	**Upper CI**	**P-value**
Age	1.044	1.031	1.056	**0.000**	1.022	1.004	1.040	**0.018**
Race: Non-White^c^	1.769	1.234	2.536	**0.002**	1.195	0.707	2.019	0.506
Gender: Male	1.108	0.738	1.661	0.621	1.105	0.658	1.854	0.706
BMI	1.048	1.017	1.079	**0.002**	0.976	0.932	1.023	0.314
BP	1.001	0.987	1.015	0.903	0.996	0.979	1.014	0.672
Chol	1.001	0.995	1.008	0.699	1.007	0.998	1.015	0.133
HDL	0.976	0.960	0.992	**0.003**	0.972	0.950	0.993	**0.010**
LDL	0.995	0.987	1.003	0.204	0.997	0.986	1.007	0.509
UA	0.833	0.722	0.961	**0.012**	0.829	0.680	1.010	0.063
TG	1.002	1.000	1.004	0.145	1.002	0.999	1.004	0.206

^a^ 95% confidence interval,

^b^ P-values are obtained based on standard Normal distribution,

^c^ Including Native American, Hispanic, and Black.

Combining these results with those in Table 7, it can be stated that the first incidence of hyperglycemia is more attributed to the diabetogenic effect of tacrolimus. However, in the absence of such an effect, the recurrent incidence of hyperglycemia is mainly imputed to other risk factors (e.g., age, race (non-White), BMI, HDL, and UA). A review of Tables 7 and 9 also shows potential consequences of choosing the right statistical tool in determining the diabetogenic effect of immunosuppressive drugs or corresponding risk factors for hyperglycemia incidence. In addition, observing that the first and recurrent types of hyperglycemia are subject to different risk factors might have broader implications for other similar chronic diseases. The current literature largely overlooks time-dependent analyses, and our results shed light on the importance of closing this gap.

### Impact of the Inpatient Period

Prior studies have addressed the importance of the inpatient period: what happens to patients during post-transplant hospitalization may have an impact on patient’s conditions after hospital discharge [67, 68]. To evaluate the impact of inpatient period, we analyzed the effect of (1) average bed glucose result (bed.avg), which is obtained by a poke test, (2) average blood glucose result (blood.avg), and (3) inpatient hyperglycemia (in.hyp) on the incidence of hyperglycemia. Table 10 summarizes the results obtained from our statistical methods. Based on Table 10, the average bed and blood glucose results are significantly associated with both the first and the recurrent incidence of hyperglycemia. However, the occurrence of inpatient hyperglycemia is only associated with recurrent incidence of hyperglycemia.

**Table 10:** Effect of inpatient period on hyperglycemia incidence.

**Inpatient Parameters**	**Part I: Time-dependent**				**Part II: Time-independent**			
	**Mean HR**	**Lower CI^a^**	**Upper CI**	**P-value^b^**	**Mean HR**	**Lower CI**	**Upper CI**	**P-value**
bed.avg^e^ (unadj^c^)	1.029	1.024	1.035	**0.000**	1.023	1.013	1.032	**0.000**
bed.avg (adj^d^)	1.024	1.018	1.030	**0.000**	1.018	1.008	1.029	**0.000**
blood.avg^f^ (unadj)	1.031	1.023	1.038	**0.000**	1.022	1.011	1.033	**0.000**
blood.avg (adj)	1.024	1.016	1.032	**0.000**	1.018	1.007	1.030	**0.002**
in.hyp^g^ (unadj)	3.509	1.557	7.908	**0.002**	2.162	0.874	5.347	0.095
in.hyp (adj)	2.496	1.080	5.768	**0.032**	1.543	0.613	3.885	0.358

^a^ 95% confidence interval,

^b^ P-values are obtained based on standard Normal distribution,

^c^ Unadjusted (univariate) analysis,

^d^ All adjusted analyses were done based on age, race, gender, BMI, BP, Chol, HDL, LDL, UA, and TG,

^e^ Average bed glucose result,

^f^ Average blood glucose result,

^g^ Inpatient hyperglycemia.

## Discussion

Our analyses highlight the complex nature of post-renal transplant hyperglycemia. Some patients never exhibit hyperglycemia, some develop permanent hyperglycemia (NODAT), while for others hyperglycemia may be transient or even recurrent. Hyperglycemia and NODAT have been mostly analyzed for a short period after transplantation [72, 73]. However, their incidence may be underestimated by such short-term studies (see [74–79] for some studies analyzing long-term analyses). Our results show that if the diabetogenic effect of immunosuppressive drugs is of interest, short-term analyses might be preferred, while long-term analyses are more suitable when studying other risk factors.

The idea of analyzing hyperglycemia from this perspective (i.e., time course of complications) can also be extended to other chronic diseases in which both the first incident and the recurrent ones need to be monitored. For example, prostate cancer and breast cancer are among diseases that may show signs only once or may do so from time to time with periods of remission in between [80, 81]. For this category of diseases, considering both time-dependent and time-independent analyses (as we did in this study) may provide new and important insights.

There are some limitations in our study. First, due to the nature of our study, having patients’ information on a more regular basis (e.g., monthly) would improve the accuracy of our results. Second, if the data set included patients’ information after the first year post-transplant, we would be able to conduct a more robust Cox regression and Kaplan-Meier survival analysis. Third, although, according to Table 5, 79 patients (who experienced post-transplant hyperglycemia for the first time) are sufficient for the purpose of our analyses, it might be a relatively small sample. Finally, even though sirolimus and steroid were used for the minority of patients (in comparison with tacrolimus), we had no information about the exact dosages and trough levels of these two drugs. Otherwise, we could also evaluate the possible association between their trough levels and incidence of hyperglycemia.

Finally, some of our findings may not be generalizable to other types of solid organ transplants (e.g., heart, liver, and pancreas). Therefore, testing our findings can be a fruitful path for future research. By extending the idea of this study and incorporating the time course of complications for other organs, one can establish a holistic framework to analyze (a) the diabetogenic effect of immunosuppressive drugs, and (b) the effect of other risk factors.

## Conclusion

We analyzed the effects of (1) immunosuppressive drugs, (2) risk factors, and (3) inpatient hyperglycemia on the first and recurrent incidence of post-transplant hyperglycemia in patients who had no history of diabetes mellitus prior to their transplants. We employed two statistical inference methods: (1) Cox regression model with time-dependent covariates to analyze hyperglycemia with recurrence and (2) Cox regression model with time-independent covariates to evaluate the first incidence of hyperglycemia. We also employed Kaplan-Meier survival analysis to characterize the cumulative probability of experiencing post-transplant hyperglycemia over time.

Based on the results obtained from these methods, we can state that the diabetogenic effect of tacrolimus (based on its trough level) can be established when hyperglycemia is experienced for the first time. However, in a sharp contrast, this effect cannot be established for the recurrent incidents of hyperglycemia. This difference might be due to the fact that tacrolimus dosage is reduced by physicians over time. As the diabetogenic effect is ruled out, our results show that age, race (non-White), BMI, HDL, steroid use, and uric acid are the only significant risk factors for the recurrent incidence.

## References

[1] Bloom RD, Crutchlow MF (2008) Transplant-Associated Hyperglycemia. Transplantation Reviews 22: 39–51. 18631857 10.1016/j.trre.2007.06.001

[2] Kesiraju S, Paritala P, Rao CUM, Sahariah S (2014) New onset of diabetes after transplantation—An overview of epidemiology, mechanism of development and diagnosis. Transplant Immunology 30: 52–58. 10.1016/j.trim.2013.10.006 24184293

[3] Räkel A, Karelis AD (2011) New-onset diabetes after transplantation: risk factors and clinical impact. Diabetes & Metabolism 37: 1–14. 10.1016/j.diabet.2010.09.003 21295510

[4] Bułanowski M, Chudek J, Wiecek A (2012) Influence of conversion from cyclosporine A to tacrolimus on insulin sensitivity assessed by euglicaemic hyperinsulinemic clamp technique in patients after kidney transplantation. Medical Science Monitor Basic Research 17: 61–68. 10.12659/aot.883459 23018257

[5] Duvoux C, Firpi R, Grazi GL, Levy G, Renner E, et al (2013) Recurrent Hepatitis C Virus Infection Post Liver Transplantation: Impact of Choice of Calcineurin Inhibitor. Transplant International 26: 358–372. 10.1111/tri.12065 23413991

[6] Furth S, Neu A, Colombani P, Plotnick L, Turner ME, et al (1996) Diabetes as a complication of tacrolimus (FK506) in pediatric renal transplant patients. Pediatric Nephrology 10: 64–66. 10.1007/BF00863448 8611359

[7] Herrero JI, Quiroga J, Sangro B, Pardo F, Rotellar F, et al (2003) Conversion from calcineurin inhibitors to mycophenolate mofetil in liver transplant recipients with diabetes mellitus. Transplantation Proceedings 35: 1877–1879. 10.1016/S0041-1345(03)00644-4 12962832

[8] Kurzawski M, Dziewanowski K, Łapczuk J, Wajda A, Droździk M (2012) Analysis of common type 2 diabetes mellitus genetic risk factors in new-onset diabetes after transplantation in kidney transplant patients medicated with tacrolimus. European Journal of Clinical Pharmacology 68: 1587–1594. 10.1007/s00228-012-1292-8 22569928 PMC3496482

[9] Levy G, Villamil F, Samuel D, Sanjuan F, Grazi GL, et al (2004) Results of lis2t, a multicenter, randomized study comparing cyclosporine microemulsion with C2 monitoring and tacrolimus with C0 monitoring in de novo liver transplantation. Transplantation 77: 1632–1638. 15201658 10.1097/01.tp.0000129095.51031.42

[10] Levy G, Grazi GL, Sanjuan F, Wu Y, Mühlbacher F, et al (2006) 12-month follow-up analysis of a multicenter, randomized, prospective trial in de novo liver transplant recipients (LIS2T) comparing cyclosporine microemulsion (C2 monitoring) and tacrolimus. Liver Transplantation 12: 1464–1472. 10.1002/lt.20802 17004259

[11] Marchetti P (2004) New-onset diabetes after transplantation. The Journal of Heart and Lung Transplantation 23: S194–S201. 10.1016/j.healun.2004.03.007 15093805

[12] Mecule A, Poli L, Nofroni I, Bachetoni A, Tinti F, et al (2010) Once daily tacrolimus formulation: monitoring of plasma levels, graft function, and cardiovascular risk factors. Transplantation Proceedings 42: 1317–1319. 10.1016/j.transproceed.2010.03.123 20534290

[13] O’grady JG, Burroughs A, Hardy P, Elbourne D, Truesdale A, et al (2002) Tacrolimus versus microemulsified ciclosporin in liver transplantation: the TMC randomised controlled trial. The Lancet 360: 1119–1125. 10.1016/S0140-6736(02)11196-2 12387959

[14] Ramachandran R, Kumar V, Rathi M, Nada R, Jha V, et al (2014) Tacrolimus therapy in adult-onset steroid-resistant nephrotic syndrome due to a focal segmental glomerulosclerosis single-center experience. Nephrology Dialysis Transplantation 29: 1918–1924. 10.1093/ndt/gfu097 24771498

[15] Saliba F, Lakehal M, Pageaux GP, Roche B, Vanlemmens C, et al (2007) Risk factors for new-onset diabetes mellitus following liver transplantation and impact of hepatitis c infection: An observational multicenter study. Liver Transplantation 13: 136–144. 10.1002/lt.21010 17192854

[16] Sharif A, Ravindran V, Moore RH, Dunseath G, Luzio S, et al (2010) Insulin Resistance Indexes in Renal Transplant Recipients Maintained on Tacrolimus Immunosuppression. Transplantation 89: 327–333. 20145524 10.1097/TP.0b013e3181bbf2c4

[17] Stevens RB, Lane JT, Boerner BP, Miles CD, Rigley TH, et al (2012) Single-dose rATG induction at renal transplantation: superior renal function and glucoregulation with less hypomagnesemia. Clinical Transplantation 26: 123–132. 10.1111/j.1399-0012.2011.01425.x 21401720

[18] Taylor AL, Watson CJE, Bradley JA (2005) Immunosuppressive agents in solid organ transplantation: Mechanisms of action and therapeutic efficacy. Critical Reviews in Oncology/Hematology 56: 23–46. 10.1016/j.critrevonc.2005.03.012 16039869

[19] Cohen EEW, Wu K, Hartford C, Kocherginsky M, Eaton KN, et al (2012) Phase I studies of sirolimus alone or in combination with pharmacokinetic modulators in advanced cancer patients. Clinical Cancer Research 18: 4785–4793. 10.1158/1078-0432.CCR-12-0110 22872575 PMC4410974

[20] Johnston O, Rose CL, Webster AC, Gill JS (2008) Sirolimus is associated with new-onset diabetes in kidney transplant recipients. Journal of the American Society of Nephrology 19: 1411–1418. 10.1681/ASN.2007111202 18385422 PMC2440303

[21] Matias P, Araujo MR, Romão JE, Abensur H, Noronha IL (2008) Conversion to Sirolimus in Kidney–Pancreas and Pancreas Transplantation. Transplantation Proceedings 40: 3601–3605. 10.1016/j.transproceed.2008.07.138 19100448

[22] Montero N, Pascual J (2015) Immunosuppression and post-transplant hyperglycemia. Current Diabetes Reviews 11 (3): 144–154. 10.2174/1573399811666150331160846 25824238

[23] Romagnoli J, Citterio F, Nanni G, Favi E, Tondolo V, et al (2006) Incidence of Posttransplant Diabetes Mellitus in Kidney Transplant Recipients Immunosuppressed with Sirolimus in Combination with Cyclosporine. Transplantation Proceedings 38: 1034–1036. 10.1016/j.transproceed.2006.03.072 16757255

[24] Teutonico A, Schena PF, Di Paolo S (2005) Glucose metabolism in renal transplant recipients: effect of calcineurin inhibitor withdrawal and conversion to sirolimus. Journal of the American Society of Nephrology 16: 3128–3135. 10.1681/ASN.2005050487 16107580

[25] Van Laecke S, Van Biesen W, Verbeke F, De Bacquer D, Peeters P, et al (2009) Posttransplantation Hypomagnesemia and Its Relation with Immunosuppression as Predictors of New-Onset Diabetes after Transplantation. American Journal of Transplantation 9: 2140–2149. 10.1111/j.1600-6143.2009.02752.x 19624560

[26] Vodenik B, Rovira J, Campistol JM (2009) Mammalian target of rapamycin and diabetes: what does the current evidence tell us? Transplantation Proceedings 41: S31–S38. 10.1016/j.transproceed.2009.06.159 19651294

[27] Bending JJ, Ogg CS, Viberti GC (1987) Diabetogenic effect of cyclosporin. BMJ 294: 401–402. 10.1136/bmj.294.6569.401 3101896 PMC1245410

[28] Borda B, Szederkényi E, Lengyel C, Morvay Z, Eller J, et al (2011) Functional and histopathologic changes in renal transplant patients with new-onset diabetes and dyslipidemia. Transplantation Proceedings 43: 1254–1258. 10.1016/j.transproceed.2011.03.091 21620104

[29] Dresner LS, Andersen DK, Kahng KU, Munshi IA, Wait RB (1989) Effects of cyclosporine on glucose metabolism. Surgery 106: 163–169. 2669194

[30] Hjelmesæth J, Hartmann A, Kofstad J, Egeland T, Stenstrøm J, et al (2001) Tapering off prednisolone and cyclosporin the first year after renal transplantation: the effect on glucose tolerance. Nephrology Dialysis Transplantation 16: 374–377. 10.1093/ndt/16.4.829 11274282

[31] Hricik DE, Bartucci MR, MOIR EJ, Mayes JT, Schulak JA (1991) Effects of steroid withdrawal on posttransplant diabetes mellitus in cyclosporine-treated renal transplant recipients. Transplantation 51: 829–835. 10.1097/00007890-199102000-00020 1994531

[32] Meerwein C, Korom S, Arni S, Inci I, Weder W, et al (2011) The Effect of Low-Dose Continuous Erythropoietin Receptor Activator in an Experimental Model of Acute Cyclosporine A Induced Renal Injury. European Journal of Pharmacology 671: 113–119. 10.1016/j.ejphar.2011.09.166 21968143

[33] Mora PF (2010) New-onset diabetes after renal transplantation. Journal of Investigative Medicine 58: 755–763. 20517164 10.231/JIM.0b013e3181e61a64

[34] Ramos-Cebrian M, Torregrosa JV, Gutierrez-Dalmau A, Oppenheimer F, Campistol JM (2007) Conversion from tacrolimus to cyclosporine could improve control of posttransplant diabetes mellitus after renal transplantation. Transplantation Proceedings 39: 2251–2253. 10.1016/j.transproceed.2007.06.035 17889154

[35] Taylor DO, Barr ML, Radovancevic B, Renlund DG, Mentzer RM Jr, et al (1999) A randomized, multicenter comparison of tacrolimus and cyclosporine immunosuppressive regimens in cardiac transplantation: decreased hyperlipidemia and hypertension with tacrolimus. The Journal of Heart and Lung Transplantation 18: 336–345. 10.1016/S1053-2498(98)00060-6 10226898

[36] Van Den Hoogen MW, Van Der Hoeven AM, Hilbrands LB (2013) Insulin requirement after a renal transplant in patients with type 2 diabetes: the choice of calcineurin inhibitors. Experimental and Clinical Transplantation 11: 234–238. 10.6002/ect.2012.0221 23432070

[37] Wyzgal J, Oldakowska-Jedynak U, Paczek L, Michalska M, Ziolkowski J, et al (2003) Posttransplantation diabetus mellitus under calcineurin inhibitor. Transplantation Proceedings 35: 2216–2218. 10.1016/S0041-1345(03)00819-4 14529893

[38] Kappe C, Fransson L, Wolbert P, Ortsater H (2015) Glucocorticoids suppress GLP-1 secretion: possible contribution to their diabetogenic effects. Clinical Science 129: 405–414. 10.1042/CS20140719 25853863

[39] Liu X, Zhu X, Miao Q, Ye H, Zhang Z, et al (2014) Hyperglycemia Induced by Glucocorticoids in Nondiabetic Patients: A Meta-Analysis. Annals of Nutrition and Metabolism 65: 324–332. 10.1159/000365892 25402408

[40] Rafacho A, Ortsäter H, Nadal A, Quesada I (2014) Glucocorticoid Treatment and Endocrine Pancreas Function. Journal of Endocrinology 223: 49–62. 10.1530/JOE-14-0373 25271217

[41] Van Genugten RE, Van Raalte DH, Muskiet MH, Heymans MW, Pouwels PJW, et al (2014) Does dipeptidyl peptidase-4 inhibition prevent the diabetogenic effects of glucocorticoids in men with the metabolic syndrome? A randomized controlled trial. European Journal of Endocrinology 170: 429–439. 10.1530/EJE-13-0610 24297090

[42] Van Raalte DH, Diamant M (2014) Steroid Diabetes: From Mechanism to Treatment? The Netherlands Journal of Medicine 72: 62–72. 24659588

[43] Wajngot A, Giacca A, Grill V, Vranic M, Efendic S (1992) The diabetogenic effects of glucocorticoids are more pronounced in low-than in high-insulin responders. Proceedings of the National Academy of Sciences 89: 6035–6039. 10.1073/pnas.89.13.6035 PMC402133 1631088

[44] Wise JK, Hendler R, Felig P (1973) Influence of glucocorticoids on glucagon secretion and plasma amino acid concentrations in man. Journal of Clinical Investigation 52: 2774–2782. 10.1172/JCI107473 4748510 PMC302545

[45] Farris AB III, Lauwers GY, Deshpande V (2010) Autoimmune pancreatitis-related diabetes: quantitative analysis of endocrine islet cells and inflammatory infiltrate. Virchows Archiv 457: 329–336. 10.1007/s00428-010-0948-y 20632032

[46] Gelens MACJ, Christiaans MHL, Van Hooff JP (2008) Glucose metabolism before and after conversion from cyclosporine microemulsion to tacrolimus in stable renal recipients. Nephrology Dialysis Transplantation 23: 701–706. 10.1093/ndt/gfm544 17999993

[47] Rajab A, Pelletier RP, Ferguson RM, Elkhammas EA, Bumgardner GL, et al (2007) Steroid-free maintenance immunosuppression with rapamune and low-dose neoral in pancreas transplant recipients. Transplantation 84: 1131–1137. 17998868 10.1097/01.tp.0000287117.98785.54

[48] Wissing KM, Pipeleers L (2014) Obesity, metabolic syndrome and diabetes mellitus after renal transplantation: Prevention and treatment. Transplantation Reviews 28: 37–46. 10.1016/j.trre.2013.12.004 24507957

[49] Carter SA, Kitching AR, Johnstone LM (2014) Four pediatric patients with autosomal recessive polycystic kidney disease developed new-onset diabetes after renal transplantation. Pediatric Transplantation 18: 698–705. 10.1111/petr.12332 25118046

[50] Gaynor JJ, Ciancio G, Guerra G, Sageshima J, Hanson L, et al (2015) Multivariable risk of developing new onset diabetes after transplant–results from a single-center study of 481 adult, primary kidney transplant recipients. Clinical Transplantation 29: 301–310. 10.1111/ctr.12510 25581205

[51] Kuo HT, Lau C, Sampaio MS, Bunnapradist S (2010) Pretransplant risk factors for new-onset diabetes mellitus after transplant in pediatric liver transplant recipients. Liver Transplantation 16: 1249–1256. 10.1002/lt.22139 21031540

[52] Luan FL, Langewisch E, Ojo A (2010) Metabolic syndrome and new onset diabetes after transplantation in kidney transplant recipients. Clinical Transplantation 24: 778–783. 10.1111/j.1399-0012.2009.01194.x 20047609 PMC3831507

[53] Lv C, Chen M, Xu M, Xu G, Zhang Y, et al (2014) Influencing factors of new-onset diabetes after a renal transplant and their effects on complications and survival rate. PloS One. 10.1371/journal.pone.0099406 PMC4050028 24911157

[54] Palepu S, Prasad GVR (2015) New-onset diabetes mellitus after kidney transplantation: Current status and future directions. World Journal of Diabetes 6: 445–455. 10.4239/wjd.v6.i3.445 25897355 PMC4398901

[55] Park SC, Yoon TD, Jung HY, Kim KH, Choi JY, et al (2015) Effect of Transient Post-transplantation Hyperglycemia on the Development of Diabetes Mellitus and Transplantation Outcomes in Kidney Transplant Recipients. Transplantation Proceedings 47: 666–671. 10.1016/j.transproceed.2014.11.053 25891707

[56] Pirsch JD, Henning AK, First MR, Fitzsimmons W, Gaber AO, et al (2015) New-Onset Diabetes After Transplantation: Results From a Double-Blind Early Corticosteroid Withdrawal Trial. American Journal of Transplantation 15: 1982–1990. 10.1111/ajt.13247 25881802

[57] Rodrigo E, Fernandez-Fresnedo G, Valero R, Ruiz JC, Pinera C, et al (2006) New-Onset Diabetes after Kidney Transplantation: Risk Factors. Journal of the American Society of Nephrology 17: 291–295. 10.1681/ASN.2006080929 17130277

[58] Parvizi Z, Azarpira N, Kohan L, Darai M, Kazemi K, et al (2014) Association between E23K variant in KCNJ11 gene and new-onset diabetes after liver transplantation. Molecular Biology Reports 41: 6063–6069. 10.1007/s11033-014-3483-0 24996284

[59] Soule JL, Olyaei AJ, Boslaugh TA, Busch AMH, Schwartz JM, et al (2005) Hepatitis C infection increases the risk of new-onset diabetes after transplantation in liver allograft recipients. The American Journal of Surgery 189: 552–557. 10.1016/j.amjsurg.2005.01.033 15862495

[60] Tokodai K, Amada N, Haga I, Nakamura A, Kashiwadate T, et al (2014) Pretransplant HbA1c Is a Useful Predictor for the Development of New-Onset Diabetes in Renal Transplant Recipients Receiving No or Low-Dose Erythropoietin. International Journal of Endocrinology. 10.1155/2014/436725 25386190 PMC4216713

[61] Wauters RP, Cosio FG, Fernandez MLS, Kudva Y, Shah P (2012) Cardiovascular consequences of new-onset hyperglycemia after kidney transplantation. Transplantation 94: 377–382. 22820698 10.1097/TP.0b013e3182584831 PMC4030430

[62] Yadav AD, Chang YH, Aqel BA, Byrne TJ, Chakkera HA, et al (2013) New onset diabetes mellitus in living donor versus deceased donor liver transplant recipients: analysis of the UNOS/OPTN database. Journal of Transplantation. 10.1155/2013/269096 24205434 PMC3800575

[63] Bayer ND, Cochetti PT, Kumar MSA, Teal V, Huan Y, et al (2010) Association of metabolic syndrome with development of new onset diabetes after transplantation. Transplantation 90: 861–866. 20724958 10.1097/TP.0b013e3181f1543c PMC2959139

[64] Lane JT, Dagogo-Jack S (2011) Approach to the patient with new-onset diabetes after transplant (NODAT). The Journal of Clinical Endocrinology & Metabolism 96: 3289–3297. 10.1210/jc.2011-0657 22058376

[65] Ghanta M, Kozicky M, Jim B, Ghanta M (2014) Pathophysiologic and Treatment Strategies for Cardiovascular Disease in End Stage Renal Disease and Kidney Transplantation. Cardiology in Review 23: 109–118. 10.1097/CRD.0000000000000044 25420053

[66] Salifu MO, Tedla F, Murty PV, Aytug S, McFarlane SI (2005) Challenges in the diagnosis and management of new-onset diabetes after transplantation. Current Diabetes Reports 5: 194–199. 10.1007/s11892-005-0009-0 15929866

[67] Chakkera HA, Knowler WC, Devarapalli Y, Weil EJ, Heilman RL, et al (2010) Relationship between Inpatient Hyperglycemia and Insulin Treatment after Kidney Transplantation and Future New Onset Diabetes Mellitus. Clinical Journal of the American Society of Nephrology 5: 1669–1675. 10.2215/CJN.09481209 20558559 PMC2974410

[68] Chakkera HA, Weil EJ Castro J, Heilman RL, Reddy KS, et al (2009) Hyperglycemia during the Immediate Period after Kidney Transplantation. Clinical Journal of the American Society of Nephrology 4: 853–859. 10.2215/CJN.05471008 19339426 PMC2666437

[69] Buuren S, Groothuis-Oudshoorn K (2011) MICE: Multivariate Imputation by Chained Equations in R. Journal of Statistical Software 45: 1–67. 10.18637/jss.v045.i03

[70] MedlinePlus Medical Encyclopedia. Available from: https://www.nlm.nih.gov/medlineplus/encyclopedia.html. Accessed: 2015-05-08.

[71] American Heart Association. Available from: http://www.heart.org/HEARTORG/Conditions/HighBloodPressure/AboutHighBloodPressure/Understanding-Blood-Pressure-Readings_UCM_301764_Article.jsp#.VivsfH6rTcs. Accessed: 2015-05-05.

[72] Chien YS, Chen YT, Chuang CH, Cheng YT, Chuang FR, et al (2008) Incidence and Risk Factors of New-Onset Diabetes Mellitus after Renal Transplantation. Transplantation Proceedings 40: 2409–2411. 10.1016/j.transproceed.2008.06.034 18790250

[73] Ghisdal L, Van Laecke S, Abramowicz MJ, Vanholder R, Abramowicz D (2012) New-Onset Diabetes after Renal Transplantation: Risk assessment and Management. Diabetes Care 35: 181–188. 10.2337/dc11-1230 22187441 PMC3241330

[74] Bee YM, Tan HC, Tay TL, Kee TY, Goh SY, Kek PC (2011) Incidence and Risk Factors for Development of New-Onset Diabetes after Kidney Transplantation. Annals Academy of Medicine Singapore 40: 160–167. 21678001

[75] Cosio FG, Pesavento TE, K, Henry ML, Ferguson RM (2001) Post-Transplant Diabetes Mellitus: Increasing Incidence in Renal Allograft Recipients Transplanted in Recent Years. Kidney International 59: 732–737. 10.1046/j.1523-1755.2001.059002732.x 11168956

[76] Davidson JA, Wilkinson A (2004) New-Onset Diabetes after Transplantation 2003 International Consensus Guidelines: An Endocrinologist’s View. Diabetes Care 27: 805–812. 10.2337/diacare.27.3.805 14988309

[77] Honda M, Asonuma K, Hayashida S, Suda H, Ohya Y et al (2013) Incidence and Risk Factors for New-Onset Diabetes in Living-Donor Liver Transplant Recipients. Clinical Transplantation 27: 426–435. 10.1111/ctr.12103 23464510

[78] Kaposztas Z, Gyurus E, Kahan BD (2011) New-Onset Diabetes after Renal Transplantation: Diagnosis, Incidence, Risk Factors, Impact on Outcomes, and Novel Implications. Transplantation Proceedings 43: 1375–1394. 10.1016/j.transproceed.2011.04.008 21693204

[79] Mozaffarian D, Marfisi R, Levantesi G, Silletta MG, Tavazzi L, et al (2007) Incidence of New-Onset Diabetes and Impaired Fasting Glucose in Patients with Recent Myocardial Infarction and The Effect of Clinical and Lifestyle Risk Factors. The Lancet 370: 667–675. 10.1016/S0140-6736(07)61343-9 17720018

[80] Cardoso F, Harbeck N, Fallowfield L, Kyriakides S, Senkus E (2012) Locally recurrent or metastatic breast cancer: ESMO Clinical Practice Guidelines for diagnosis, treatment and follow-up. Annals of Oncology 23: vii11–vii19. 10.1093/annonc/mds232 22997442

[81] Mohler JL, Gregory CW, Ford OH, Kim D, Weaver CM, et al (2004) The androgen axis in recurrent prostate cancer. Clinical Cancer Research 10: 440–448. 10.1158/1078-0432.CCR-1146-03 14760063

